# The prevalence and correlates of self-harm in pregnant women with psychotic disorder and bipolar disorder

**DOI:** 10.1007/s00737-016-0636-2

**Published:** 2016-05-13

**Authors:** Clare L. Taylor, Leontien M. van Ravesteyn, Mijke P. Lambregtse van denBerg, Robert J. Stewart, Louise M. Howard

**Affiliations:** 1 Department of Health Services and Population Research, Institute of Psychiatry, Psychology and Neuroscience, King’s College London, London, UK; 2 Department of Psychiatry, Erasmus University Medical Center, Rotterdam, The Netherlands; 3 Department of Child & Adolescent Psychiatry, Erasmus University Medical Center, Rotterdam, The Netherlands; 4 Department of Psychological Medicine, Institute of Psychiatry, Psychology and Neuroscience, King’s College London, London, UK; 5 Section of Women’s Mental Health, PO31 Institute of Psychiatry, Psychology & Neuroscience, King’s College London, De Crespigny Park, SE5 8AF London, UK

**Keywords:** Self-harm, Psychosis, Bipolar disorder, Pregnancy

## Abstract

Women with severe mental illness are at increased risk of suicide in the perinatal period, and these suicides are often preceded by self-harm, but little is known about self-harm and its correlates in this population. This study aimed to investigate the prevalence of suicidal ideation and self-harm, and its correlates, in women with psychotic disorders and bipolar disorder during pregnancy. Historical cohort study using de-identified secondary mental healthcare records linked with national maternity data. Women pregnant from 2007 to 2011, with ICD-10 diagnoses of schizophrenia and related disorders, bipolar disorder or other affective psychoses were identified. Data were extracted from structured fields, natural language processing applications and free text. Logistic regression was used to examine the correlates of self-harm in pregnancy. Of 420 women, 103 (24.5 %) had a record of suicidal ideation during the first index pregnancy, with self-harm recorded in 33 (7.9 %). Self-harm was independently associated with younger age (adjusted odds ratio (aOR) 0.91, 95 % CI 0.85–0.98), self-harm in the previous 2 years (aOR 2.55; 1.05–6.50) and smoking (aOR 3.64; 1.30–10.19). A higher prevalence of self-harm was observed in women with non-affective psychosis, those who discontinued or switched medication and in women on no medication at the start of pregnancy, but these findings were not statistically significant in multivariable analyses. Suicidal thoughts and self-harm occur in a significant proportion of pregnant women with severe mental illness, particularly younger women and those with a history of self-harm; these women need particularly close monitoring for suicidality.

## Background

The perinatal period is generally a time of both lower suicide risk (Appleby 1991; Marzuk et al. 1997) and lower self-harm risk (Appleby and Turnbull 1995; Weiss 1999), but for women with severe mental disorders (SMI), the risk of suicide is increased up to 70-fold in women admitted for postpartum psychiatric disorders (Appleby et al. 1998). The UK Confidential Enquiries into Maternal Deaths and other studies have highlighted mental illness as a significant contributor to maternal deaths and also highlight a history of self-harm in a significant proportion (25–50 %) of maternal suicides (Kurinczuk JJ 2014). Compared with the postnatal period, women who die by suicide during pregnancy are reported more likely to have a diagnosis of schizophrenia/related disorders or of bipolar disorder and less likely to have a diagnosis of depression (Khalifeh et al. 2016). Little is known about the prevalence and risk factors of self-harm in pregnant women with severe mental disorders even though self-harm in pregnancy is potentially harmful to the viability of the pregnancy in addition to being a potential risk factor for suicide. Risk factors for self-harm in the general population include a history of self-harm (Tidemalm et al. 2014), younger age (Moran et al. 2012), substance misuse (Hawton et al. 2002), domestic and sexual violence (Khalifeh et al. 2015), genetic risk (Finseth et al. 2014) and severity of illness (Mauri et al. 2013). In addition, in pregnancy, a recent study (Zhong et al. 2015) showed younger age and depression diagnoses were risk factors for suicidal behaviour-related hospitalisations in pregnant women but did not look at other mental health diagnoses. Illness severity and relapse have been associated with discontinuation of medication in one small study in women with bipolar disorder (Viguera et al. 2007), but associations with risk of self-harm remain under-investigated.

We therefore aimed to investigate the prevalence of suicidal ideation and self-harm in pregnant women with SMI (schizophrenia/related disorders, bipolar disorder and other affective psychoses). We hypothesised that self-harm would be associated with markers of illness severity (non-affective diagnosis, substance misuse, smoking, a recent history of self-harm, recent hospitalisation), younger age, discontinuation or switching of regular maintenance psychotropic medication and recent domestic violence.

## Methods

### Study design and data source

Historical cohort study uses de-identified electronic health records. Pregnant women with SMI were identified using the South London and Maudsley (SLaM) NIHR Biomedical Research Centre Clinical Record Interactive Search (CRIS) system (Stewart et al. 2009). This is a “new generation” of case register design, built on full electronic clinical records and allowing in-depth secondary analysis of both numerical, string and free-text data, while preserving anonymity through technical and procedural safeguards (Fernandes et al. 2013). It is a rich source of prospective clinical data. SLaM provides near-monopoly mental healthcare for a geographic catchment of around 1.2 million residents across four London boroughs, as well as specialist services. CRIS was approved as a source of the secondary data for research by Oxfordshire Research Ethics Committee C (08/H0606/71+5).

Fully electronic health records have been maintained since 2006, and at the time of data extraction, over 200,000 individuals had received care from SLaM. Several natural language processing applications have been developed for CRIS using General Architecture for Text Engineering (GATE) software in collaboration with Sheffield University (Cunningham et al. 2013). Such applications derive structured data from free-text fields. As part of CRIS development, a data linkage service has been set up to link CRIS with other sources of secondary data, including Hospital Episode Statistics (HES) which provide national statistical data for all treatment in National Health Service hospitals in England, and includes maternity data.

### Study population

This study is part of a larger programme of research on a cohort of pregnant women with SMI, described in detail previously (Taylor et al. 2015). The cohort includes all pregnant women with a diagnosis of schizophrenia and other non-affective psychoses, bipolar disorder, other affective psychoses including psychotic depression or previous puerperal psychosis (ICD-10 F20, F22, F23, F25, F28, F29, F30, F31, F32.3, F33.3 and F53.1), receiving SLaM care between 2007 and 2011. We excluded women with no SLaM clinical data during pregnancy. Structured fields and a GATE software application were used to extract the diagnosis nearest to the beginning of pregnancy.

### Measures

We used a free-text search for records of suicidal ideation (SI) and self-harm during the first index pregnancy occurring in the study cohort. Self-harm was defined as a suicide attempt or self-injurious behaviour, including cutting, burning, hitting, hanging, overdosing, poisoning and electrocuting using terms validated in another CRIS study on self-harm and Emergency Department attendances (Polling et al. 2015). Complete notes on the self-harm event were scanned for further information on the method of self-harm, triggers (reported hallucinations around 24 h of the event, alcohol and substance use within 12 h of the event) and location (whether event occurred on an inpatient ward or at home and whether the patient was under intensive Home Treatment).

Some socio-demographic variables were extracted from structured fields (age, ethnicity) and others from free text (partner status in index pregnancy). Free-text searches were also used for domestic violence before and/or during pregnancy and a history of child abuse. Smoking, alcohol and substance abuse during pregnancy were extracted using free-text searches and/or recent diagnosis of an alcohol or substance use disorder. Measures of illness nature/severity were affective/non-affective SMI diagnosis at the beginning of pregnancy, self-harm and admissions to acute care in the previous 2 years. The highest total Health of the Nation Outcome Scale (HoNOS) score in the 2 years before pregnancy was extracted to approximate baseline level of functioning. HoNOS, a structured instrument, is a 12-item measure of health and social functioning of people with severe mental illness, routinely collected in UK mental health services; scores above 10 indicate poor functioning and are generally recorded in inpatients (Wing et al. 1998). Information on acute admissions (including inpatient and intensive Home Treatment) was extracted from CRIS and HES (Taylor et al. 2015). Home Treatment Teams are a national network of teams providing intensive community-based support as an alternative to hospital admission for acutely unwell patients (Johnson et al. 2008). Regular psychotropic medication use (antidepressants, mood stabilisers or antipsychotics) and changes in the first trimester were also extracted: psychotropic drug names and changes in regular use of these medication groups during first trimester were extracted using GATE software to guide retrieval of clinical text (Taylor et al. 2015). Where no drugs were identified, a free-text search for “medication” was used, and where it was not possible to establish whether medication was being used or not in the first trimester, this was coded as “not known.” Regular maintenance medication in the first trimester was categorised into “stopped or switched a medication,” “continued a medication” and “no medication at the beginning of pregnancy.” “Non-adherence” was coded if there was a comment in the notes regarding concern about adherence indicating the possibility of no exposure to a given medication in the first trimester. For self-harm and medication changes occurring in the first trimester, we checked the notes manually to ascertain which happened first: the self-harm or the medication change.

### Data-analysis

We used Stata version 12 (Statacorp. 2011). Independent-sample *T* tests and Mann-Witney tests (for continuous data) and Pearson’s chi-square (for categorical data) were used to compare demographic and clinical characteristics between women with or without a record of self-harm during pregnancy. Cells containing *n* < 5 were not reported to maintain anonymity of the data. Cases with missing information on presence of a partner, reported abuse, substance misuse, current smoking and self-harm history were assumed to indicate that these were not present. Inter-rater agreement on self-harm data was assessed for the first 10 records and for a 10 % random sample; two raters agreed on 89 % of the data and consensus meetings resolved discrepancies; detailed guidance on how to code data was used for subsequent data coding.

Multivariable logistic regression analysis using cases with known medication status was performed to examine the correlates of self-harm during pregnancy. We compared these women with those with missing medication status on age, ethnicity, baseline diagnosis and acute admissions (variables in the multivariable analysis that did not depend solely on clinical text). HoNOS was not entered into the multivariable model as data were only available for 236 of 420 women. We conducted a sensitivity analysis excluding women who had a self-harm event before a medication change in the first trimester in order to address the potential issue of reverse causality. We also conducted sensitivity analyses using women who stopped medication only compared with continuers and excluding women reported as non-adherent to medication. Selection was based on our a priori variables and those with *p* ≤ 0.2 in the bivariate analysis. All hypothesis testing was two-tailed with *α* set to 0.05.

## Results

Our study population consisted of 420 women. Of these, 40 women had pregnancies which ended in the first trimester and 10 in the second. HoNOS scores were available for 236 women only, and 413 women had data on medication status in the first index pregnancy. There was no other missing data on covariates.

### Prevalence of suicidal ideation and self-harm in the index pregnancy, *n* = 420 women

For 103 (24.5 %) women, there was a report of suicidal ideation during the index pregnancy, while 178 (42.4 %) women denied this; in the remaining 139 (33.1 %) women, there was no mention of suicidal ideation or self-harm. For 70 (16.7 %) women, there was a report of suicidal ideation but there was no event of self-harm reported during the pregnancy. Of the 420 women in the study, 33 (7.9 %) had a self-harm event during their index pregnancy, and 9 of these had multiple events (range 1–7); 15 women had a self-harm event in the first trimester, 16 in the second trimester and 10 in the third.

### Self-harm by event, *n* = 52 events

In total, 52 events of self-harm (but no suicides) were reported in 33 women out of 420 (1 event per 19 pregnancies). Of the 52 events, methods of self-harm were overdoses (*n* = 20, 38.5 %), hitting (*n* = 12, 23.1 %), cutting (*n* = 9, 17.3 %) or using a violent method (*n* = 11, 21.2 %) such as jumping from height, burning or hanging. Of 52 self-harm events, 23 (43.1 %) occurred while women experienced hallucinations. In 18 out of 52 (34.6 %) events, drugs or alcohol were involved within 12 h before the self-harm. The majority of self-harm events took place at home (*n* = 38, 73.1 %) compared with an inpatient setting (*n* = 14, 26.9 %). Of events that took place at home (*n* = 43), 13 (30.2 %) were carried out while the woman was under intensive Home Treatment care.

### Factors associated with self-harm during the index pregnancy, *n* = 420 women

All women with self-harm in the first index pregnancy also reported suicidal ideation in pregnancy. Self-harm in pregnancy was associated with younger age, a history of child abuse or domestic violence, current (i.e. during pregnancy) domestic violence, a history of self-harm in the 2 years preceding pregnancy, substance misuse, smoking, non-affective disorder, acute admissions in the 2 years preceding pregnancy and stopping or switching rather than continuing a maintenance medication in the first trimester of pregnancy (Table 1).

**Table 1:** Baseline characteristics of 420 pregnant women with SMI

	Whole sample, *N* = 420	387 women without self-harm	33 women with a self-harm event	*p* value
Age, mean (SD)^T^	31.9 (6.2)	32.3 (6.1)	27.6 (5.5)	<0.001*
Ethnicity, *N* (%)				
African Caribbean, other black background	209 (49.8)	193 (49.9)	16 (48.5)	0.283
White British, other white background	136 (32.4)	128 (33.1)	8 (24.2)	
Mixed, unknown and other background	75 (17.9)	66 (17.1)	9 (27.3)	
Has partner during pregnancy, *N* (%)	272 (64.8)	250 (64.6)	22 (66.7)	0.811
Child abuse or DV before pregnancy, *N* (%)	191 (45.5)	170 (43.9)	21 (63.6)	0.029*
DV in pregnancy, *N* (%)	82 (19.5)	70 (18.1)	12 (36.4)	0.011*
Suicidal ideation in pregnancy, *N* (%)	103 (24.5)	70 (18.1)	33 (100.0)	>0.001*
Self-harm 2 years before pregnancy, *N* (%)	62 (14.8)	47 (12.1)	15 (45.5)	<0.001*
Harmful use of alcohol or substances, *N* (%)	107 (25.5)	88 (22.7)	19 (57.6)	<0.001*
Smoking in pregnancy, *N* (%)	76 (18.1)	58 (15.0)	18 (54.6)	<0.001*
Baseline diagnosis, *N* (%)				
Non-affective	219 (52.1)	194 (50.1)	25 (75.8)	<0.005*
Affective	201 (47.9)	193 (49.9)	8 (24.2)	
Hospitalisation or home treatment within 2 years before pregnancy, *N* (%)	180 (42.9)	158 (40.8)	22 (66.7)	0.004*
HoNOS, median (range), *N* = 236^W^	12 (0,36)	12 (0,36)	14 (3,28)	0.090
Antipsychotic or mood stabiliser, 1st trimester, *N* (%), *n* = 413	277 (67.1)	250 (65.8)	27 (81.8)	0.060
Antidepressant, 1st trimester, *N* (%), *n* = 413	99 (24.0)	91 (24.0)	8 (24.2)	0.970
Medication change, 1st trimester, *N* (%) *n* = 413				
Continuation of previous agent	168 (40.7)	162 (42.6)	6 (18.2)	Ref
Stopped/switched agent	117 (28.3)	101 (26.6)	16 (48.5)	0.003*
No medication at start of pregnancy	128 (31.0)	117 (30.8)	11 (33.3)	0.074
Medication change, 1st trimester sensitivity analysis, *N* = 409				
Continued	168 (41.1)	162 (42.6)	6 (20.7)	Ref
Stopped/switched	115 (28.1)	101 (26.6)	14 (48.3)	0.009*
No medication at start of pregnancy	126 (31.0)	117 (30.8)	9 (31.0)	0.177

*SMI* severe mental illness, *DV* domestic violence

*p < 0.05

^T^Independent-sample *T* tests

^W^Mann-Whitney test

For multivariable analyses, 7 women were excluded as their medication status in the first trimester of pregnancy was not known. Therefore, 413 women were included, 33 (8.0 %) of whom had a self-harm event in their index pregnancy. Suicidal ideation was not included in the analyses due to perfect prediction. There were no differences in age (*t* = −0.21, *p* = 0.833), ethnicity (chi^2^ = 4.53, *p* = 0.104), diagnosis (chi^2^ = 1.59, *p* = 0.208) and admission rate (chi^2^ = 0.00, *p* = 1.000) between those who were included and not included in the multivariable analyses.

In the fully adjusted models (Table 2), there was evidence of associations between self-harm in pregnancy and younger age, smoking and a recent history of self-harm and the adjusted odds ratio for discontinuation or change in maintenance medication compared with continuing medication was attenuated by around 50 % and no longer significant.

**Table 2:** Multivariate models of socio-demographic and clinical characteristics in women who self-harm in pregnancy

	Whole sample		Excluding women with medication changes after self-harm events in 1st trimester		Excluding women with non-adherence to medication		Excluding women who switched medication	
	Unadjusted *N* = 413	Fully adjusted *N* = 413	Unadjusted *N* = 409	Fully adjusted *N* = 409	Unadjusted *N* = 393	Fully adjusted *N* = 393	Unadjusted *N* = 388	Fully adjusted *N* = 388
Age	0.88 (0.83,0.94) <0.001*	0.91 (0.85,0.98) 0.009*	0.88 (0.82,0.94) <0.001*	0.91 (0.84,0.97) 0.008*	0.87 (0.82,0.93) <0.001*	0.91 (0.84,0.97) 0.006*	0.90 (0.84,0.96) 0.001*	0.93 (0.86,1.00) 0.043*
Child abuse or DV before pregnancy	2.23 (1.07,4.67) 0.033*	1.31 (0.55,3.11) 0.536	1.81 (0.84,3.89) 0.130	1.12 (0.46,2.73) 0.801	2.17 (1.03,4.45) 0.042*	1.37 (0.57,3.26) 0.482	2.43 (1.06,5.55) 0.035*	1.46 (0.57,3.71) 0.427
DSH in 2 years before pregnancy	5.90 (2.79,12.50) <0.001*	2.55 (1.05,6.50) 0.039*	5.00 (2.25,11.13) <0.001*	2.30 (0.90,5.86) 0.082	5.46 (2.54,11.74) <0.001*	2.30 (0.93,5.70) 0.071	6.69 (2.95,15.16) <0.001*	3.02 (1.17,7.84) 0.023*
Harmful substance use	4.50 (2.17,9.35) <0.001*	1.68 (0.61,4.59) 0.314	4.08 (1.89,8.82) <0.001*	1.67 (0.58,4.83) 0.342	4.37 (2.09,9.17) <0.001*	1.53 (0.54,4.29) 0.264	4.00 (1.80,8.87) 0.001*	1.67 (0.58,4.82) 0.343
Smoking in pregnancy	6.66 (3.18,13.96) <0.001*	3.64 (1.30,10.19) 0.014*	5.95 (2.73,12.98) <0.001*	3.36 (1.13,9.97) 0.029*	6.89 (3.24,14.65) <0.001*	3.81 (1.32,10.99) 0.013*	5.99 (2.67,13.44) <0.001*	3.48 (1.17,10.35) 0.025*
Baseline diagnosis (non-affective)	3.06 (1.35,6.96) 0.008*	2.29 (0.91,5.71) 0.077	3.75 (1.49,9.43) 0.005*	2.66 (0.98,7.20) 0.055	3.02 (1.32,6.89) 0.009*	2.18 (0.86,5.49) 0.099	2.94 (1.21,7.12) 0.017*	2.15 (0.81,5.72) 0.125
Admitted in 2 years before pregnancy	2.90 (1.37,6.16) 0.005*	1.87 (0.79,4.42) 0.153	2.76 (1.25,6.09) 0.012*	1.87 (0.77,4.55) 0.168	2.81 (1.32,6.01) 0.008*	1.65 (0.69,3.98) 0.264	2.53 (1.13,5.69) 0.024	1.63 (0.66,4.08) 0.292
Medication change								
Continued	Ref	Ref	Ref	Ref	Ref	Ref	Ref	Ref
Stopped/switched	4.28 (1.62,11.29) 0.003*	2.48 (0.84,7.31) 0.099	3.74 (1.39,10.05) 0.009*	2.26 (0.75,6.77) 0.145	4.23 (1.59,11.29) 0.004*	2.55 (0.85,7.65) 0.095	3.29 (1.16,9.38) 0.026*	1.90 (0.58,6.17) 0.287
No medication at start of pregnancy	2.54 (0.91,7.06) 0.074	2.36 (0.76,7.32) 0.137	2.08 (0.72,5.99) 0.177	1.95 (0.60,6.26) 0.265	2.43 (0.87,6.77) 0.088	2.17 (0.70,6.71) 0.180	2.54 (0.91,7.06) 0.074	2.33 (0.75,7.23) 0.142

*p < 0.05

Fewer than five women had a medication change after the self-harm event. Sensitivity analysis excluding these women led to a further attenuation of the relationship between medication changes and self-harm (Table 2), and the association with history of self-harm was no longer significant. Non-affective diagnosis appeared to be associated with self-harm, but this did not quite reach statistical significance (*p* = 0.055). Other sensitivity analyses did not lead to substantial differences (see Table 2).

## Discussion

This is the first study, to our knowledge, to report on the prevalence of self-harm in pregnant women with severe mental illness, a group already recognised to be at increased risk of maternal suicide. We found that 25 % of this cohort had recorded suicidal ideation in pregnancy, a similar rate to that reported in a clinical population of 360 pregnant women referred to a perinatal mental health programme with lifetime history of DSM Axis I mental disorder (Newport et al. 2007). Of particular, clinical concern was our finding of self-harm in 8 %, with violent methods used in a fifth, indicating potential severity of intent and illness which has been found in studies of suicide in general populations (Windfuhr and Kapur 2011) and perinatal suicides (Khalifeh et al. 2016). We confirmed our hypotheses that self-harm was associated with illness severity—smoking, and previous self-harm and, though weaker evidence, non-affective diagnosis.

Medication discontinuation was not significantly associated with self-harm, though this may reflect a lack of statistical power. In pregnancy, clinicians as well as patients may be concerned to avoid medication due to concerns about teratogenicity, particularly in the first trimester, and we have described elsewhere that 78.6 % of those who stopped medication in this cohort were indeed recorded as stopping “because of the pregnancy” (Taylor et al. 2015). Recent systematic reviews and well-designed cohort studies suggest that the small increased risk of congenital malformations in this population appears to be due to confounding factors, (NICE 2014; Khalifeh et al. 2015) other than for some mood stabilisers, particularly valproate (Meador et al. 2006). While this study cannot confirm whether or not medication changes in pregnancy could lead to self-harm, there are likely to be complex relationships between illness severity, medication change and self-harming behaviour that are not easy to disentangle in observational research. Screening and close monitoring is therefore essential for pregnant women with SMI, particularly those with markers of severity who discontinue medication, in order to prevent repetition of self-harm (Moran et al. 2012).

Finally, it was noteworthy that this population had high prevalence of substance misuse, smoking and reported domestic abuse—all risk factors for adverse foetal outcomes which need to be addressed by maternity and mental health professionals (Stein et al. 2014).

### Strengths and limitations

Strengths of the study include the use of data from a large representative sample of pregnant women with SMI observed longitudinally within a comprehensive clinical database. The unique features of the data source enabled us to access data on a group who are a particularly hard-to-reach population to recruit into clinical studies (Woodall et al. 2011). Other studies looking at self-harm, suicide and suicidal ideation in pregnancy have often excluded patients with SMI; utilising these clinical records enabled us to capture a group of women who are potentially at particular risk and yet are under-represented in research, particularly women with schizophrenia. The use of Hospital Episode Statistics provided a robust method of identifying pregnancies regardless of hospital of birth. It did not include home births which account for about 2.4 % of births for England in 2011 (ONS 2013); however, as women with SMI are high-risk pregnancies, it is unlikely that many deliver at home. Hospital Episode Statistics also enabled us to collect information on admission histories covering the whole of England for this potentially mobile population. An additional strength was the detailed information on psychotropic medication use, in addition to histories of abuse and other exposures usually not available in administrative datasets.

Limitations include the use of information recorded by clinical staff, which could underestimate suicidal ideation and self-harm prevalence—women may not disclose suicidal thoughts or acts as they may be worried about custody loss (Dolman et al. 2013; Megnin-Viggars et al. 2015). There was no mention of suicidal ideation in the notes for 33 % of women, which may have been due to suboptimal record-keeping. Finally, we cannot assume generalisability to all women with SMI and pregnancy as we did not include women with SMI managed solely in primary care.

## Conclusions

The comparatively high level of suicidal ideation reported, and the significant levels of self-harm recorded, indicates that women with SMI in pregnancy are a high-risk population who require close monitoring in pregnancy.
